# Evaluation of *Escherichia coli* drug resistance of relict gull (*Larus relictus*) in Hongjian Nur, Shaanxi, China

**DOI:** 10.3389/fvets.2026.1783278

**Published:** 2026-03-19

**Authors:** Anwen He, Dingding Wu, Na Zhao, Nan Wang, Ping Feng, Junjun Zhai, Yan Zhang

**Affiliations:** 1 College of Advanced Agricultural Sciences, Yulin University, Yulin, Shaanxi, China; 2 Yulin City Key Laboratory of Veterinary Public Health and Biosafety, Yulin University, Yulin, Shaanxi, China; 3 Shaanxi Cashmere Goat Engineering Technology Research Center, Yulin University, Yulin, Shaanxi, China

**Keywords:** drug resistance gene, *Escherichia coli*, Hongjian Nur, relict gull, resistance phenotype

## Abstract

Antibiotic resistance (AMR) is one of today’s most pressing global public health crises, which effective containment of its potential negative effects requires regular monitoring of bacterial resistance. Wild birds are recognized as reservoirs and potential transmitters of antibiotic resistance, thus playing a critical role in the dissemination of resistant bacteria. Therefore, we evaluated the resistance phenotypes and resistance genes of *E. coli* in the intestinal tract of relict gulls in the Hongjian Nur area of Shaanxi Province. In this study, fecal samples from relict gulls were collected for *E. coli* isolation and identification, drug susceptibility testing, drug resistance gene detection, phylogenetic clustering, and multi-locus sequence typing. A total of 172 *E. coli* strains were isolated. The results of the drug susceptibility tests indicated phenotypic resistance rates of 37.21% for azithromycin, 12.21% for ampicillin, and 9.88% for tetracycline, with 19.95% of the strains exhibiting multiple drug resistance. PCR amplification results showed that the carrying rates of the first five resistance genes were all above 90%, with resistance rates for *tem* and *strA* both at 99.42%. The phylogenetic clustering of multi-drug resistant *E. coli* predominantly belonged to the B2 group. Sequence typing revealed that several *E. coli* alleles were primarily ST4162, ST1299, ST1196, ST297, and ST2570. Our findings indicate a serious level of drug resistance among *E. coli* from relict gulls, characterized by a high proportion of multiple drug resistance and a relatively high detection rate of various antibiotic resistance genes. By further investigating the distribution of drug-resistant bacteria in relict gulls, this study provides foundational data for understanding the resistance phenotypes and distribution of drug-resistant genes in migratory birds, thereby contributing to efforts aimed at alleviating the increasingly severe global antibiotic resistance issue.

## Background

Migratory animals are integral to ecosystem biodiversity, and their biological activities contribute to the functioning and stability of ecosystems. Among migratory species, birds have received substantial attention as potential vectors of pathogens because they can travel long distances within short time periods (1–4). Birds that are closely associated with urban habitats and other areas of human activity are more likely to carry drug-resistant *Escherichia coli* (*E. coli*), thereby providing transmission routes between urban and natural environments (5). Concurrently, scientists speculate that migratory birds may serve as reservoirs for drug-resistant bacteria and resistance genes, facilitating their spread to various regions across the globe (6). Consequently, assessing the epidemiological role of migratory birds as vectors of drug resistance is essential for evaluating ecosystem health, reflecting human activities and their impacts in a timely manner (7, 8).

Relict gulls (*Larus relictus*) are endangered migrant birds belonging to a European family of medium-sized waterfowl. They primarily inhabit desert saltwater lakes, and alkaline lakes at altitudes of 1,200–1,500 meters, thriving under desert and semi-desert ecological conditions. These birds migrate north in March and south in October each year (9). Recognized as an effective species in 1971, relict gulls were subsequently listed in Appendix I of the Convention on International Trade in Endangered Species of Wild Fauna and Flora (CITES) and the Convention on Migrant Species (CMS). They are categorized as a threatened species in the “Red Book” published by the International Union for Conservation of Nature (IUCN). As a rare and endangered species globally, relict gulls are also classified as a Class I protected bird in China (10).

*Escherichia coli* is found in the intestines of humans and various animal species, including birds, and is recognized as one of the most prevalent zoonotic pathogens (11). It is frequently utilized as an indicator bacterium for assessing antimicrobial selective pressure in laboratory settings. *E. coli* is frequently utilized as an indicator bacterium for assessing antimicrobial selective pressure in laboratory settings (12). Furthermore, *E. coli* not only acquires drug resistance genes from other bacteria but also disseminates these genes to other bacterial strains through various mechanisms, enabling horizontal gene transfer among different bacterial types (13). Among the reported pathogens affecting wild birds, *E. coli* is the most significant (4).

Currently, drug resistant microorganisms have emerged as one of the most significant health challenges in both public health and veterinary medicine (14). The rapid spread of multi-drug-resistant bacteria has increasingly propelled humanity into what is referred to as the “post-antibiotic era” (15). *E. coli*, being the most widely distributed bacterium, is frequently monitored as a key indicator of antibiotic resistance due to its inherent characteristics (16). During the surveillance of *E. coli* resistance, numerous resistance genes have been identified as being closely linked to both human and animal health (17). Additionally, *E. coli* isolates from wild birds across various regions of the world exhibit commonalities with other host-associated resistant pathogens (18–20). Notably, the majority of bacterial pathogens that produce extended-spectrum *β*-lactamases (Esbl) in wild birds are *E. coli* (21). Islam et al. (22) found that migratory birds in Bangladesh carry high levels of *E. coli* resistance. Nearly 40% of *E. coli* isolates produce extended-spectrum beta-lactamases, and all of these are multi resistant strains, with resistance to tetracycline, fluoroquinolones, and streptomycin being the most prevalent. This finding aligns with the research conclusions of Guenther et al. (23) regarding common wild birds in Europe. The abuse of antibiotics has accelerated the emergence of multi resistance; however, the mechanisms of antibiotic resistance and the distribution of resistance genes within wild bird populations remain inadequately understood (24). Investigating antibiotic resistance and gaining a comprehensive understanding of resistant bacteria and resistance gene distribution in wild birds are essential for designing effective prevention strategies against the threat of antibiotic resistance. Therefore, this study aimed to determine the *E. coli* resistance phenotypes and antibiotic resistance gene profiles. *E. coli* was isolated from relict gulls feces collected in Shenmu City, Shaanxi Province, and its resistance to 15 different antibiotics and the presence of 49 resistance genes were assessed. This research is significant for the conservation of species diversity, the assessment of clinical safety, the prediction and early warning of drug-resistant strain risks, and the safeguarding of public health.

## Materials and methods

### Sample collection and bacterial isolation

In this study, 67 fecal samples from relict gulls were randomly collected in the Hongjian Nur area of Shenmu City, stored at low temperatures, and subsequently sent to the laboratory. In the laboratory, the stool samples were placed in centrifuge tubes, to which an appropriate volume of 0.9% normal saline was added, and the mixture was thoroughly ground. *E. coli* was isolated from the feces using McConkey solid medium. Subsequently, a single colony was picked from the McConkey agar and Gram-stained according to established protocols, with the results observed under an oil immersion microscope. After confirmation via staining and microscopic examination, the single colonies were enriched in LB liquid medium for further research. All media were incubated at 37 °C for 18 to 24 h to facilitate bacterial growth.

### Bacterial DNA extraction

For bacterial DNA extraction, 1 mL of pure LB culture colonies was selected according to the instructions provided in the bacterial genomic DNA extraction kit. The supernatant obtained during centrifugation was stored at −20 °C and utilized as template DNA.

### *Escherichia coli* molecular bioassay

The *E. coli* housekeeper gene icd was amplified via PCR using the pure bacterial solution DNA from the isolated strain as a template, allowing for the determination of the *E. coli* genotype. The primer sequences were as follows: icd (F): ATGGAAAGTAAAGTAGTTGTTCCGGCACA; icd (R): GGACGCAGCAGGATCTGTT, with the 878 relict gulls band being considered positive. The PCR mixture was prepared with a total volume of 10 μL, comprising 5 μL of 2 × Taq PCR premix reagent II, 0.5 μL of each primer, 1 μL of template DNA, and 3 μL of ddH2O. The PCR amplification conditions included an initial denaturation step at 94 °C for 5 min, followed by 30 cycles of denaturation (30 s at 94 °C), annealing (30 s at 54 °C), and extension (30 s at 72 °C), concluding with a final extension at 72 °C for 10 min. After mixing the amplified PCR products with loading dyes, 10 μL of the mixture was transferred using a micropipette and loaded onto a 1.5% agarose gel. The mixture was then validated by gel electrophoresis at 120 V for 25 min and observed using an ultraviolet (UV) transilluminator. The *E. coli* strain ATCC 25922 was provided by Professor Zhu Zhanbo of Heilongjiang Bayi Agricultural University as a quality control strain, while mixtures without template DNA served as negative controls.

### Antimicrobial susceptibility testing

Isolates were assessed for resistance to commonly used antibiotics using the Kirby-Bauer disk diffusion method, in accordance with the protocols outlined in the CLSI guidelines. The selection of antibiotics was primarily based on their prevalent use and significance to both public and animal health. Ultimately, the antibiotic groups (*β*-lactams, aminoglycosides, quinolones, lincomycin, macrolides, colistin, chloramphenicol, cephalosporins, sulfonamides, and tetracyclines) and specific antibiotics (imipenem, ampicillin, cefazolin, gentamicin, streptomycin, tetracycline, enrofloxacin, norfloxacin, lincomycin, azithromycin, chloramphenicol, florfenicol, cefquinome, fonomide, and polymyxin B) were selected.

The bacterial suspension for the drug susceptibility test was prepared in advance. In a sterile environment, 100 μL of the bacterial suspension was inoculated onto LB solid medium, which was evenly coated using a coater. Drug susceptibility tablets were then uniformly placed onto the medium using tweezers and appropriately labeled. The plates were inverted and incubated at 37 °C for 16–18 h. The results were interpreted by measuring the diameter of the inhibition zone according to CLSI guidelines and recorded as resistant (R), intermediate (I), or sensitive (S). Bacterial isolates that exhibited resistance to at least three antibiotics from different classes (≥3 antibiotic groups) were classified as multidrug-resistant (MDR) isolates.

### Antibiotic resistance genes determination

Using specific primers, single PCR was employed to identify antibiotic resistance genes. This study focused on the resistance genes conferred to *E. coli* by antibiotics such as quinolones, tetracyclines, sulfonamides, and aminoglycosides. Based on the resistance genes, primers, and annealing temperatures (as outlined in Supplementary Table 1), the presence of *E. coli* resistance genes in relict gulls was assessed. The PCR was conducted in a reaction mixture with a total volume of 10 μL, with the composition mirroring the PCR fractions specified in the *E. coli* Molecular Bioassay. Amplification conditions were tailored according to the annealing temperature and the length of the target fragment. The amplified products were subjected to electrophoresis on a 1.5% agarose gel, and the results were observed and documented.

### Phylogenetic clustering of *Escherichia coli* and MLST

According to the method reported by Tang et al. (25), the *chuA*, *yiaA*, and *tspE4.C2* genes (Table 1) were detected simultaneously using single PCR. Based on the various combinations of *chuA*, *yiaA*, and *tspE4.C2*, *E. coli* can be categorized into four populations: Group A (*chuA*−, *TspE4.C2*−), Group B1 (*chuA*−, *tspE4.C2*+), Group B2 (*chuA*+, *yiaA*+), and Group D (*chuA*+, *yiaA*−). Screening multidrug resistant *E.coli* Strains from Drug Susceptibility Test for Multilocus Sequence Typing (MLST). Primers were synthesized based on seven housekeeping genes, including *adk*, *fumC*, *gyrB*, *icd*, *mdh, purA*, and *recA*, as listed in the *E. coli* MLST database^1^ (Table 1) (26, 27). These primers were employed to amplify the genes of multidrug-resistant strains, and the resulting products were sequenced. The sequencing results were analyzed using the ChromasPro software, and the outcomes were subsequently uploaded to the MLST database^2^ to identify the corresponding sequence number and ST type.

**Table 1:** *Escherichia coli* clustering and housekeeping gene primers.

Gene	Primer	Nucleotide sequence (5′–3′)	Amplicon size (bp)	Tm (°C)
chuA	chuA-F	GACGAACCAACGGTCAGGAT	279	57
	chuA-R	TGCCGCCAGTACCAAAGACA		
yiaA	yiaA-F	TGCCGCCAGTACCAAAGACA	211	57
	yiaA-R	ATGGAGAATGCGTTCCTCAAC		
TspE4.C2	TspE4.C2-F	GAGTAATGTCGGGGCATTCA	152	57
	TspE4.C2-R	CGCGCCAACAAAGTATTACG		
adk	adk-F	ATTCTGCTTGGCGCTCCGGG	583	52
	adk-R	CCGTCAACTTTCGCGTATTT		
fumC	fumC-F	TCACAGGTCGCCAGCGCTTC	806	52
	fumC-R	TACGCAGCGAAAAAGATTC		
gyrB	gyrB-F	TCGGCGACACGGATGACGGC	911	58
	gyrB-R	ATCAGGCCTTCACGCGCATC		
icd	icd-F	ATGGAAAGTAAAGTAGTTGTTCCGGC	878	54
	icd-R	GGACGCAGCAGGATCTGTT		
mdh	mdh-F	AGCGCGTTCTGTTCAAATGC	932	60
	mdh-R	CAGGTTCAGAACTCTCTCTGT		
purA	purA-F	CGCGCTGATGAAAGAGATGA	816	54
	purA-R	CATACGGTAAGCCACGCAGA		
recA	recA-F	CGCATTCGCTTTACCCTGACC	780	58
	recA-R	TCGTCGAAATCTACGGACCGGA		

### Statistical analysis

The laboratory results were analyzed descriptively using SPSS. A chi-square test was employed to assess the relationship between the carriage of specific resistance genes and the expression of phenotypic resistance. Statistical significance was determined at a *p*-value of less than 0.05.

## Results

### Isolation and molecular bioassay of *Escherichia coli*

**Figure 1:**
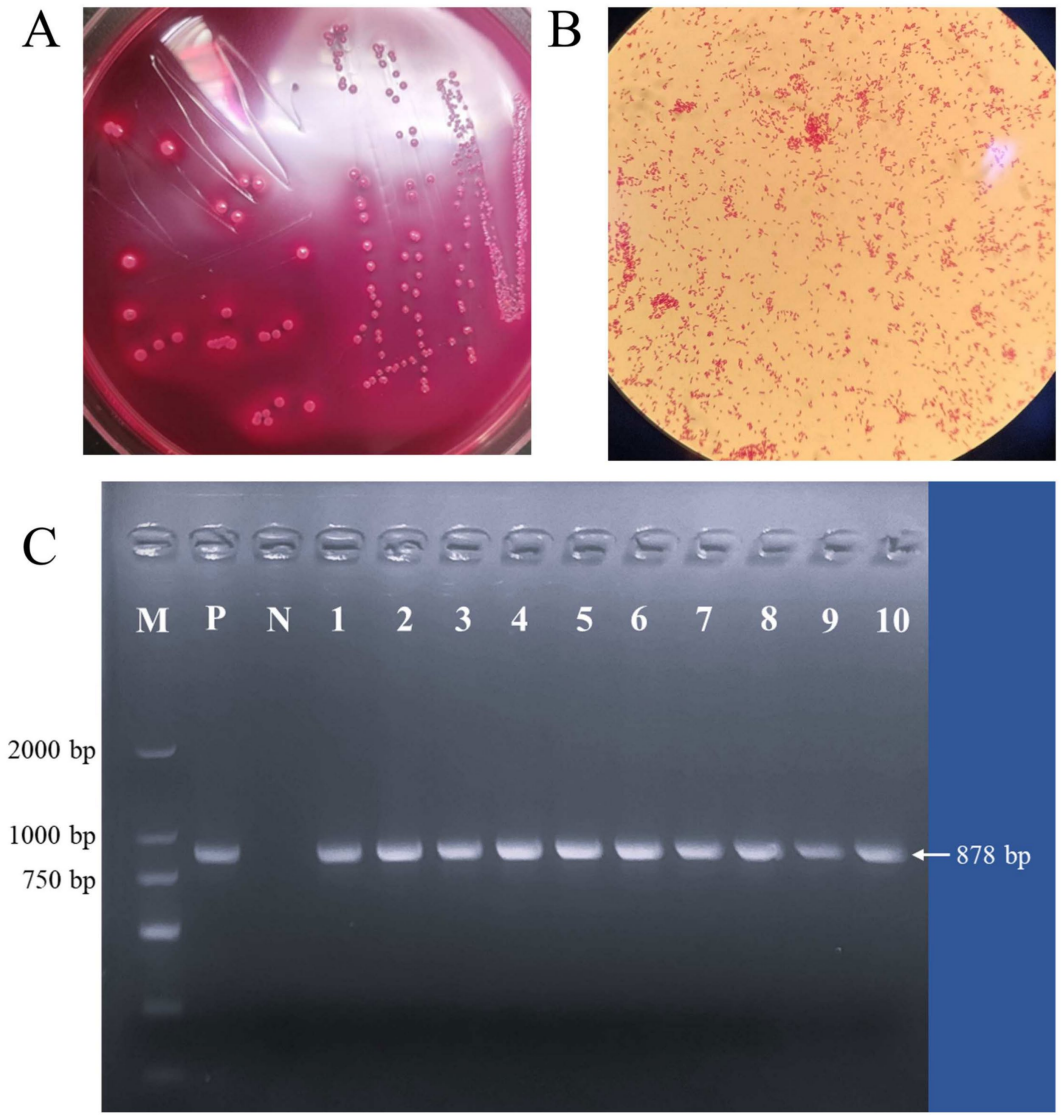
Isolation and identification of *E. coli*: **(A)** Growth on McConkey medium; **(B)** observation of Gram staining under a microscope; **(C)** identification of *E. coli* (M: Marker; P: Positive control; N: Negative control; 1–10: Isolate strains).

A total of 172 *E. coli* strains were isolated using morphological and molecular biological identification methods. The colonies appeared fresh peach red or reddish on the medium, with a dark peach red center. They were round, flat, and characterized by neat edges, a smooth surface, and a moist texture (Figure 1A). Gram staining revealed that the bacteria were rod-shaped and stained red, confirming their classification as Gram-negative bacteria (Figure 1B). PCR amplification was conducted using *E. coli*-specific primer icd, resulting in a pre-amplified fragment length of 878 bp (Figure 1C).

### Antimicrobial susceptibility testing

**Figure 2:**
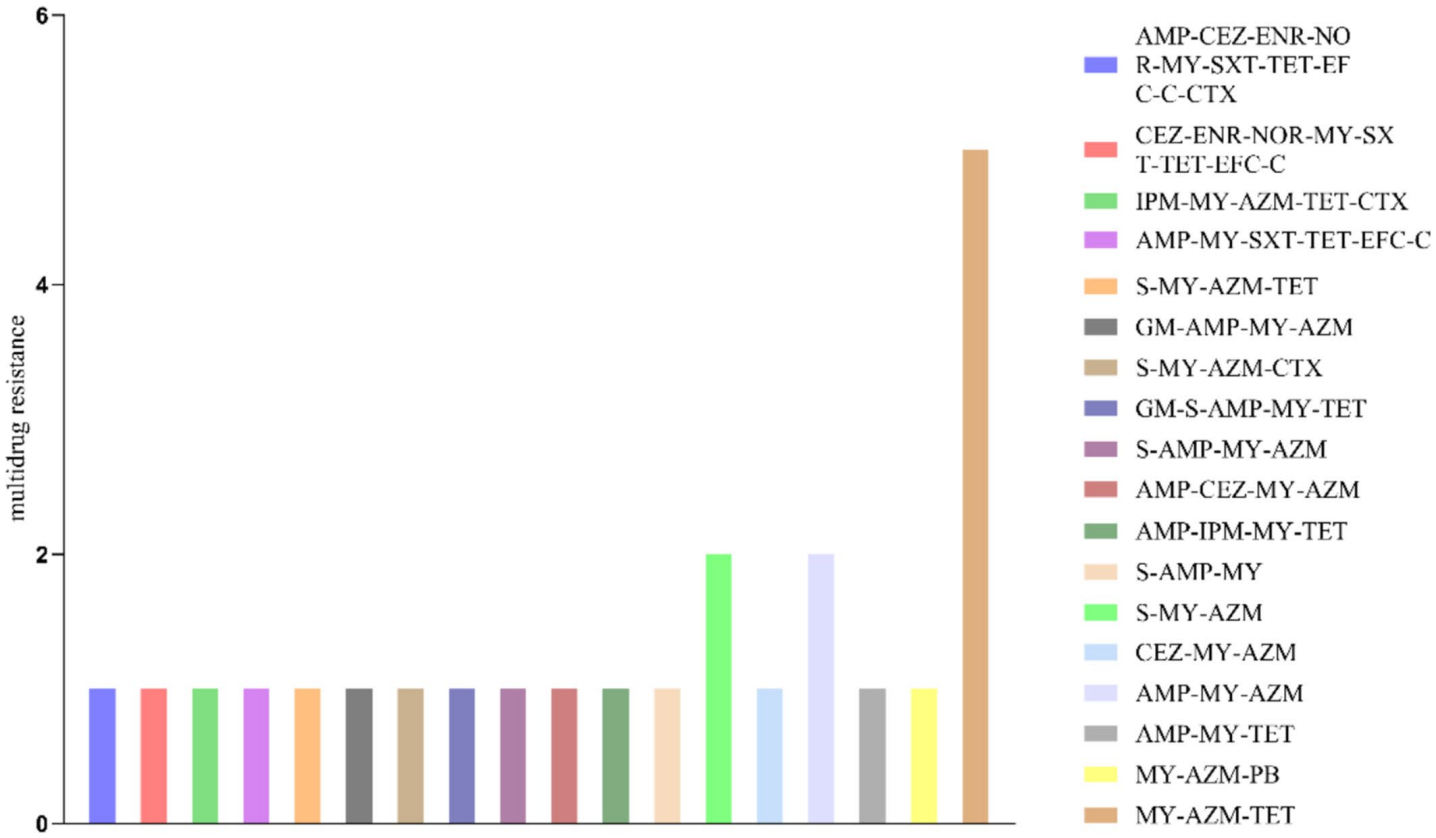
Illustrates the results of the multidrug resistance of *E. coli* isolated from relict gulls. The vertical axis represents the number of *E. coli* strains, while the horizontal axis depicts the combinations of phenotypic resistance, including GM (Gentamicin), S (Streptomycin), AMP (Ampicillin), CEZ (Cefazolin), IPM (Imipenem), ENR (Enrofloxacin), NOR (Norfloxacin), MY (Lincomycin), SXT (Paediatric Compound Sulfamethoxazole Tablets), AZM (Azithromycin), TET (Tetracycline), PB (Polymyxin B), EFC (Florfenicol), C (Chloramphenicol), and CTX (Cefquinome).

The drug susceptibility test results indicated (as illustrated in Figure 2 and Table 2) that among the 172 *E. coli* strains examined, 64 (37.21%) were resistant to azithromycin, 21 (12.21%) to ampicillin, 17 (9.88%) to tetracycline, 10 (5.81%) to streptomycin, 7 (4.07%) to cefazolin, 6 (3.49%) to imipenem, and 4 (2.33%) to enrofloxacin. Furthermore, 1.74% of the isolates exhibited phenotypic resistance to florfenicol, polymyxin B, chloramphenicol, cefquinoxime, fonomine, and norfloxacin. Notably, all but one isolate (171 strains, or 99.42%) were found to be resistant to lincosamines. Additionally, it was observed that 19.95% of *E. coli* strains displayed multiple drug resistance (as shown in Figure 2), with triple drug resistance accounting for 62.5% (15/24), quadruple drug resistance for 20.8% (5/24), quintuple drug resistance for 8.3% (2/24), six-drug resistance for 4.2% (1/24), and seven-drug resistance for 4.2% (1/24).

**Table 2:** Results of phenotypic drug susceptibility tests of *E. coli* isolated from relict gulls.

Antibiotic	Antibiotic abbreviations	*n* = 172		
		Resistance (%)	Intermediary (%)	Sensitivity (%)
Aminoglycoside	GM	2 (1.16)	17 (9.88)	153 (88.95)
	S	10 (5.81)	40 (23.26)	122 (70.93)
Beta-lactam	AMP	21 (12.21)	38 (22.09)	113 (65.70)
	CEZ	7 (4.07)	12 (6.98)	153 (88.95)
	IPM	6 (3.49)	6 (3.49)	160 (93.02)
Fluoroquinolone	ENR	4 (2.33)	15 (8.72)	153 (88.95)
	NOR	3 (1.74)	2 (1.16)	167 (97.09)
Lincosamide	MY	171 (99.42)	0 (0.00)	1 (0.58)
Sulfonamide	SXT	3 (1.74)	2 (1.16)	167 (97.09)
Macrolide	AZM	64 (37.21)	51 (29.65)	57 (33.14)
Tetracycline	TET	17 (9.88)	89 (51.74)	66 (38.37)
Colistin	PB	3 (1.74)	5 (2.91)	164 (95.35)
Phenicols	EFC	3 (1.74)	14 (8.14)	155 (90.12)
	C	3 (1.74)	8 (4.65)	161 (93.60)
Cephalosporin	CTX	3 (1.74)	42 (24.42)	127 (73.84)

### Antibiotic resistance genes determination

**Figure 3:**
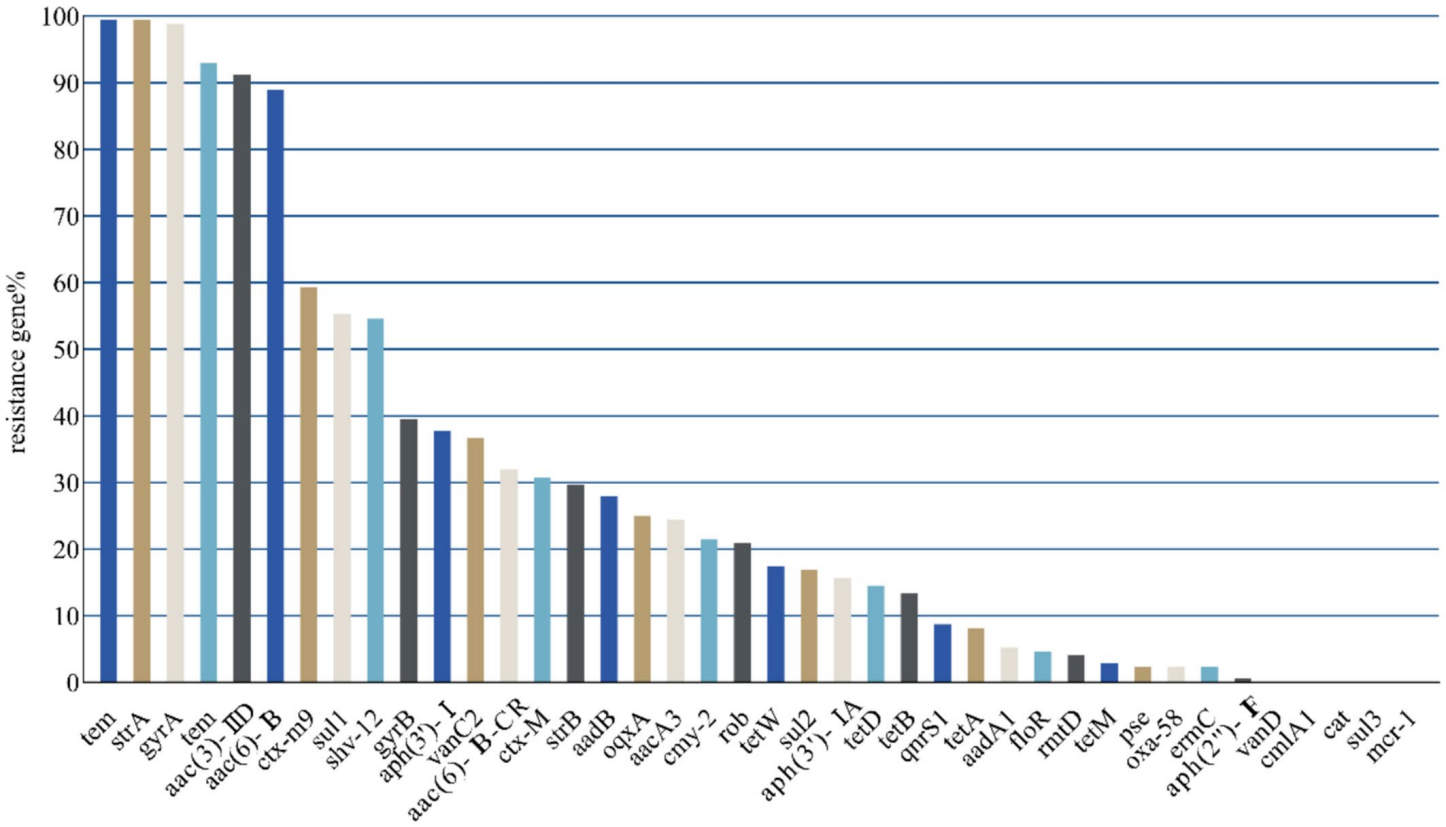
Gene detection results (horizontal axis: resistance genes; longitudinal axis: percentage in 172 *E. coli* strains).

A total of 40 resistance genes were identified in 172 *E. coli* strains obtained from relict gulls. The detection rates of the *tem* and *strA* genes were assessed through PCR amplification, with both genes present in 171 strains (99.42%) (Figure 3). The positive rates for *gyrA*, *tem*, *aac(3)-IID*, and aac(6)-*IB* were slightly lower, found in 170 (98.84%), 160 (93.02%), 157 (91.28%), and 153 (88.95%) strains, respectively. Notably, more than 90% of the 172 *E. coli* strains carried the first five resistance genes. Both *tem* primer and *tem-1* primer, which are classified as beta-lactam resistance genes, were detected in 160 *E. coli* strains (Supplementary Figures 1, 2). The *sul1* gene was identified in 95 isolates (55.23%), although most sulfonamide-resistant genes were found to be combined genes. The *sul3* gene was not detected in any of the 172 *E. coli* isolates from relict gulls, and only four isolates exhibited simultaneous detection of both *sul1* and *sul2*. Tetracycline resistance gene determinants, *tetA* and *tetB*, were found in 14 (8.14%) and 23 (13.37%) *E. coli* isolates, respectively (Figures 3 and Supplementary Figure 3). Among the chloramphenicol resistance genes, only *floR* was detected, accounting for 14 strains (4.65%), while neither *cmlA1* nor *cat* was identified. Furthermore, the 172 *E. coli* strains from relict gulls harbored a significant number of resistance genes, with each strain carrying at least five resistance genes; notably, 29 *E. coli* strains (16.86%) possessed 15 or more resistance genes.

### Correlation between resistance gene and phenotype

From the correlation of phenotypic and genotypic resistance profiles, 23.52% (4/17) and 35.29% (6/17) of tetracycline-resistant strains carried *tetA* and *tetD*, respectively. The carrying of these genes were significantly associated with tetracycline phenotypic resistance (*p* < 0.05). All *E. coli* isolates with tetracycline phenotypic resistance had *teA*, *tetD* or both. Similarly, there was a significant correlation between *ctx-M* gene carrying and cefazolin (*p* < 0.05, *X^2^* = 5.65). Two *E. coli* strains with phenotypic resistance to cefazolin did not have ctx-M gene, but had both tem. The rest were found in cefazolin resistant strains (12) and (153), and 48 strains had ctx-M gene (Table 3).

**Table 3:** Relationship between phenotype and genotype of relict gull *E. coli.*

Drug group and resistance genes		Number of isolates carrying the resistance gene		*X*^2^	*p*-value
		Resistant^a^	Non-resistant^b^		
Cefazolin	*ctx-M*	5 (71.43%)	48 (29.09%)	5.65	0.047
Ampicillin	*cmy-2*	0 (0.00%)	37 (24.50%)	6.556	0.023
Tetracycline	*tetA*	4 (23.52%)	10 (6.45%)	5.976	0.048
Tetracycline	*tetD*	6 (35.29%)	19 (12.26%)	6.544	0.028

a Isolates that are phenotypically resistant to the indicated antibiotic and carry the resistance gene.

b Isolates phenotypically susceptible or intermediately resistant to the indicated antibiotic but carry the resistance gene.

### Phylogenetic clustering of *Escherichia coli* and MLST

**Figure 4:**
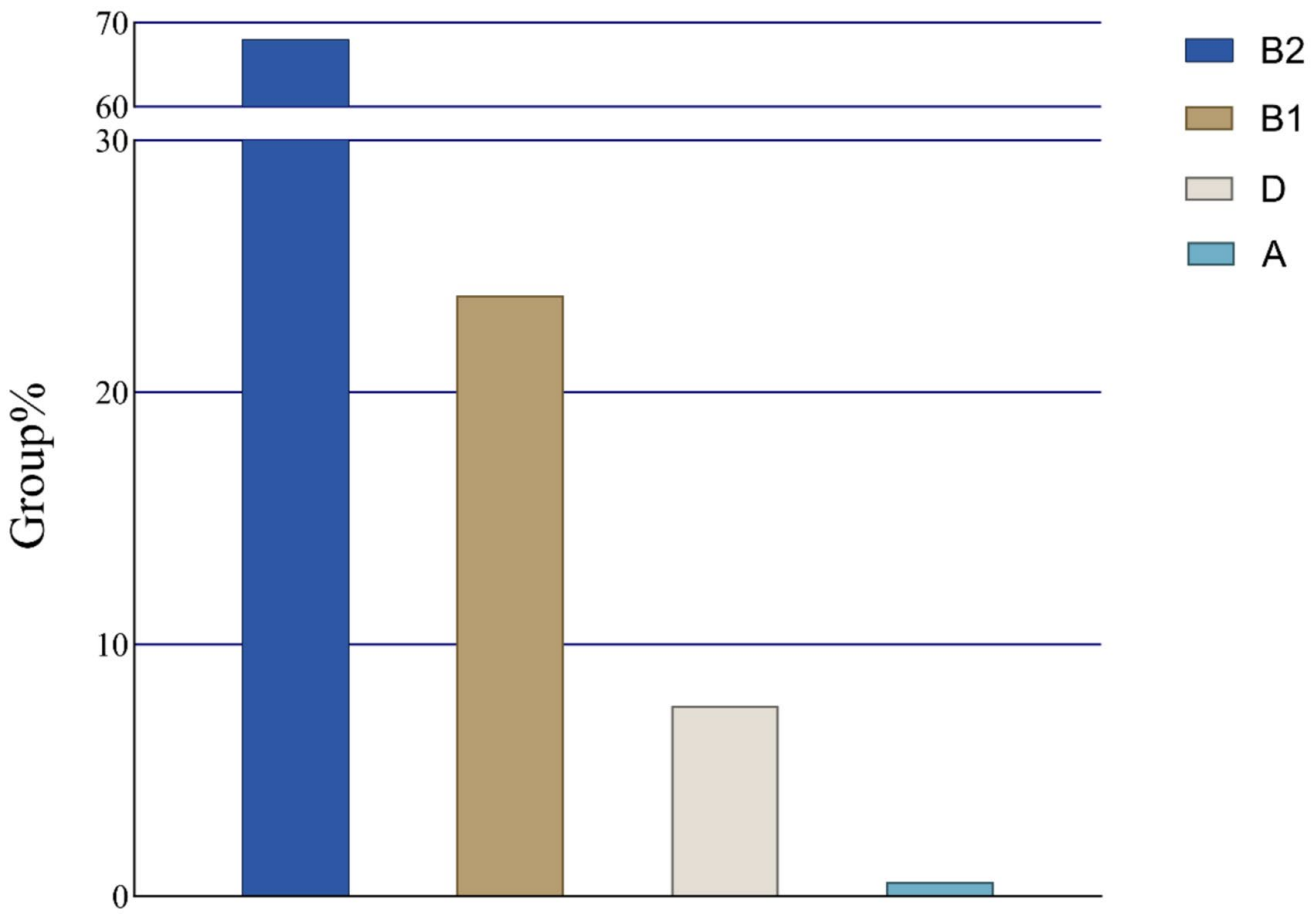
Results of evolutionary clustering of *E. coli* system.

**Figure 5:**
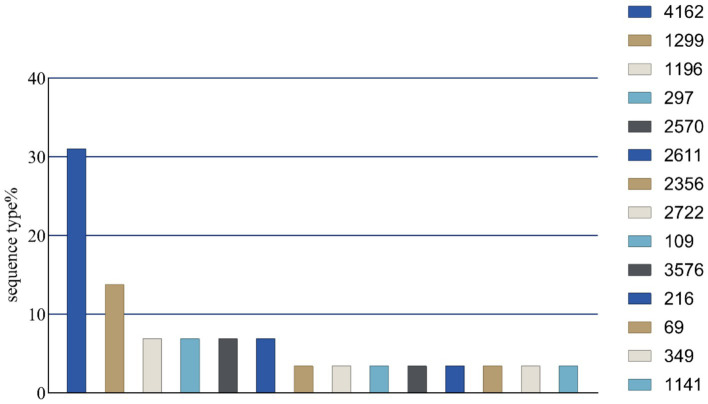
Multidrug-resistant *E. coli* genotype results.

A total of 172 *E. coli* strains were amplified for different bands of the *chuA*, *yiaA*, and *TspE4.C2* genes (Supplementary Figure 4). The results indicated that there were 117 strains (68.02%) belonging to the *E. coli* group B2 and 41 strains (23.84%) in group B1. Additionally, 13 strains (7.56%) were identified in group D, while only one strain (0.58%) was classified in group A (Figure 4). Seven housekeeping gene fragments of the expected size were amplified from 24 *E. coli* strains exhibiting multiple resistances, alongside 5 strains demonstrating the highest number of resistance genes. Sequence comparison results revealed that 29 strains of multidrug-resistant *E. coli* (MDR *E. coli*) were categorized into 14 sequence types (STs), with 9 strains (31.03%) identified as ST4162, the most prevalent type. ST1299 was observed in 4 strains (13.79%) of *E. coli*, making it the second most common after ST4162. Furthermore, ST1196, ST297, ST2570, and ST2611 were each detected in 2 strains (6.09%) of *E. coli*, while 1 strain (6.09%) represented the remaining STs (Figure 5).

## Discussion

Antimicrobial resistance (AMR) is a major global threat to human and animal health and is projected to cause up to 10 million deaths annually by 2050 if not effectively controlled (20, 28). Wild birds serve as sentinels for the dissemination of antimicrobial resistance. Widespread use of antibiotics produces a large amount of wastewater and garbage, and relict gulls often inhabit aquatic environments affected by human activities, which may contribute to the spread of drug-resistant bacteria in different geographical areas (29). A study conducted in Russia revealed that wild birds exhibit high levels of resistance to critical antibiotics, such as extended-spectrum cephalosporins, fluoroquinolones, colistin, and carbapenems (30). Therefore, monitoring migratory birds is essential, as they represent a significant pathway for the spread of bacterial resistance, warranting attention for potential risks associated with this issue.

This study investigated the resistance phenotype and genotype of *E. coli* in the gut of gulls. A total of 172 isolates of *E. coli* were subjected to drug sensitivity tests, revealing that 95.35% of the isolates were resistant to polymyxin B, which aligns with findings from Kwaśna et al. (31). Phenotypic resistance was observed for while azithromycin (37.21%), ampicillin (12.21%), and tetracycline (9.88%) also exhibited some levels of resistance. Among the 172 strains of *E. coli*, multidrug resistance was found in 19.95% of the isolates. Zhao et al. (32) reported on *E. coli* sampled from Dalian Bathing Beach, where drug resistance testing indicated a high resistance rate to tetracycline (24.6%). The multidrug resistance rate for *E. coli* in that study was 58%, which differs from our findings, this discrepancy may be attributed to differences in sample sources and sizes. Furthermore, studies from various regions worldwide have documented high multidrug resistance rates ranging from 60 to 100% (33–35). The findings of Turkey (36) and Li et al. (37) are consistent with our results. These findings confirm that *E. coli* exhibits high resistance to lincosamide antibiotics while showing relatively lower resistance to ampicillin.

Tetracyclines play a crucial role in treating various infections in animals and are categorized as “highly important antimicrobial agents” for human infections (20). Studies on wild bird resistance conducted globally have reported high rates of tetracycline resistance, ranging from 40 to 80% (2, 38, 39). In contrast, the detection rate of tetracycline resistance gene was 9.88%, which differs significantly from recent studies on tetracycline resistance in *E. coli* (40). Given the significance of tetracycline in clinical treatment, the relatively low resistance rate observed in the current study may be attributed to the predominant breeding of sheep in the local area. Previous research indicated that *E. coli* derived from sheep demonstrates relatively high resistance to tetracycline, which could be linked to local animal treatment practices, thereby resulting in reduced transmission of tetracycline resistance genes in China.

The results of the tetracycline resistance gene detection indicated a close relationship between the presence of tetracycline resistance genes *tetA* and *tetB* and tetracycline resistance itself. The positive detection rate of *tetB* among tetracycline-resistant strains was found to be 13.37%, which slightly differs from the drug resistance levels reported by Wang et al. (41) This discrepancy may be attributed to variations in regional backgrounds and distinct drug-resistant flora. Additionally, a study conducted by Fashae et al. (42) found that 85 and 18% of tetracycline-resistant *E. coli* isolates possessed the *tetA* and *tetB* genes, respectively.

The resistance genes for eight different antibiotics were detected through PCR amplification. The results indicated a relatively high detection rate for aminoglycosides, with positive detection rates of *strA* (99.42%), *aac(3)-IID* (91.28%), and *aac(6)-IB* (88.92%), all exceeding 80%. This finding is consistent with the detection rates of aminoglycoside resistance genes in *E. coli* from sheep reported by Gu et al. (43). This suggests that *E. coli* possesses a significant ability to harbor drug resistance genes and can facilitate the spread of drug resistance genes among various animal populations. The inappropriate use of antibiotics may lead to the horizontal transfer of drug resistance genes, thereby posing a threat to public health. Notably, the positive detection rates for *tem-1* primer in beta-lactams (99.42%) and *tem* primer (93.02%) also exceeded 90%. Furthermore, Yu et al. (15) noted a slight increase in the detection of *tem β*-lactamase resistance genes. In contrast, the positive rates for other classes, including glycopeptides, quinolones, macrolides, and chloramphenicol, were lower than those of the aforementioned four classes. By assessing the degree of resistance to various antibiotics, this study enhances the accuracy and effectiveness of antibiotic treatment, thereby reducing unnecessary antibiotic use and mitigating the risk of drug resistance.

Drug resistance genes can confer resistance to specific antibiotics in bacteria. The results presented in Table 3 indicate that certain drug resistance genes in *E. coli* are associated with antibiotic resistance. A significant association was observed between tetA/tetD and tetracycline resistance (*p* ≤ 0.05), whereas most other genes showed no statistical correlation, highlighting the complexity of genotype–phenotype relationships. Additionally, gene editing and other technological advancements may provide valuable insights into the molecular mechanisms underlying bacterial drug resistance and offer potential strategies for mitigating this issue in the future.

*E. coli* phylogenetic groups are categorized into A, B1, B2, and D based on their pathogenicity and drug resistance. Groups A and B1 primarily consist of symbiotic *E. coli*, whereas groups B2 and D contain a higher prevalence of pathogenic *E. coli*. In this study, most isolates belonged to phylogroup B2, and the detection rate of drug-resistant genes is notably high, suggesting the presence of potentially virulent *E. coli* lineages in relict gulls. These findings underscore the ecological relevance of migratory birds in the environmental maintenance of antimicrobial resistance. Among the 29 multidrug-resistant *E. coli* isolates, 14 distinct sequence types (STs) were identified, with ST4162 being the predominant lineage. The predominance of ST4162 among MDR isolates warrants further surveillance to clarify its ecological and epidemiological significance. Although ST4162 has been reported in human-associated contexts, its detection in relict gulls should be interpreted within a One Health framework rather than as evidence of direct transmission. In a study conducted by Martínez-Álvarez et al. (44) in Spain, the ST genotype carrying the *blaCTX-M-1* resistance gene was isolated from the cloaca samples of white stork nestlings. This finding aligns with the results of the current experiment, although the relic gulls exhibited a greater variety of resistance genes. In contrast, poultry-derived *E. coli* in Henan exhibited distinct ST distributions and markedly higher resistance levels than those observed in relict gulls, likely reflecting stronger antimicrobial selection pressure in intensive production systems (45). ST1196 has been reported in both clinical and animal-associated settings and is known to carry extended-spectrum *β*-lactamase and carbapenemase genes, indicating potential clinical relevance (46–49). Additionally, ST297, belonging to the O25b: H4 ST131 clonal complex, represents a globally disseminated antibiotic-resistant lineage frequently associated with human infections (50). The identification of these STs in relict gulls highlights the ecological connectivity between wildlife and human-associated bacterial populations.

Antibiotic-resistant bacteria in wild birds are generally associated with indirect exposure to anthropogenic contamination, such as wastewater discharge and livestock runoff, rather than direct antimicrobial treatment (42). Variations in environmental management practices may therefore influence resistance prevalence across regions. This study has several limitations. First, although 67 fecal samples were collected (yielding 172 *E. coli* isolates), the sample size and sampling frame may not fully capture temporal variability in resistance patterns within the relict gull population. Second, sampling was restricted to a single geographical location (Hongjian Nur, Shenmu City, Shaanxi Province), which limits the generalizability of the results to other breeding or migratory stopover sites where antibiotic exposure pressures may differ. Third, methodological constraints may have influenced resistance estimates: disk diffusion testing provides phenotypic categorization but can vary by breakpoint interpretation and does not quantify MICs. Finally, fecal sampling and culture-based isolation may underrepresent non-culturable bacteria or strains present at low abundance. Future work multi-site, multi-season surveillance incorporating MIC testing and genome-based approaches would strengthen inference on sources and transmission pathways.

## Conclusion

This study shows that wild relict gulls in the Hongjian Nur region carry antimicrobial-resistant *E. coli*, including multidrug-resistant isolates. The detection of multiple resistance determinants and diverse sequence types suggests that migratory birds may contribute to the maintenance and environmental dissemination of antimicrobial resistance at the wildlife–environment–human interface. These findings support incorporating migratory birds into One Health AMR surveillance, and further work is needed to clarify the sources and transmission pathways of resistance in this system.

## Data Availability

The original contributions presented in the study are included in the article/supplementary material, further inquires can be directed to the corresponding author/s.

## Footnotes

1
https://enterobase.warwick.ac.uk/species/index/ecoli

2
https://pubmlst.org/escherichia
