# Supplementary material for: Evaluation of Escherichia coli drug resistance of relict gull (Larus relictus) in Hongjian Nur, Shaanxi, China
Source: Front Vet Sci. 2026 Mar 19;13:1783278. doi: 10.3389/fvets.2026.1783278 (PMC13043378; doi:10.3389/fvets.2026.1783278)
Supplement: Supplementary file 1 [file Supplementary_Data_Sheet__1.DOCX]

Supplementary Material

# Supplementary Figures and Tables

For more information on Supplementary Material and for details on the different file types accepted, please see [here](https://www.frontiersin.org/guidelines/author-guidelines#supplementary-material).

## Supplementary Figures


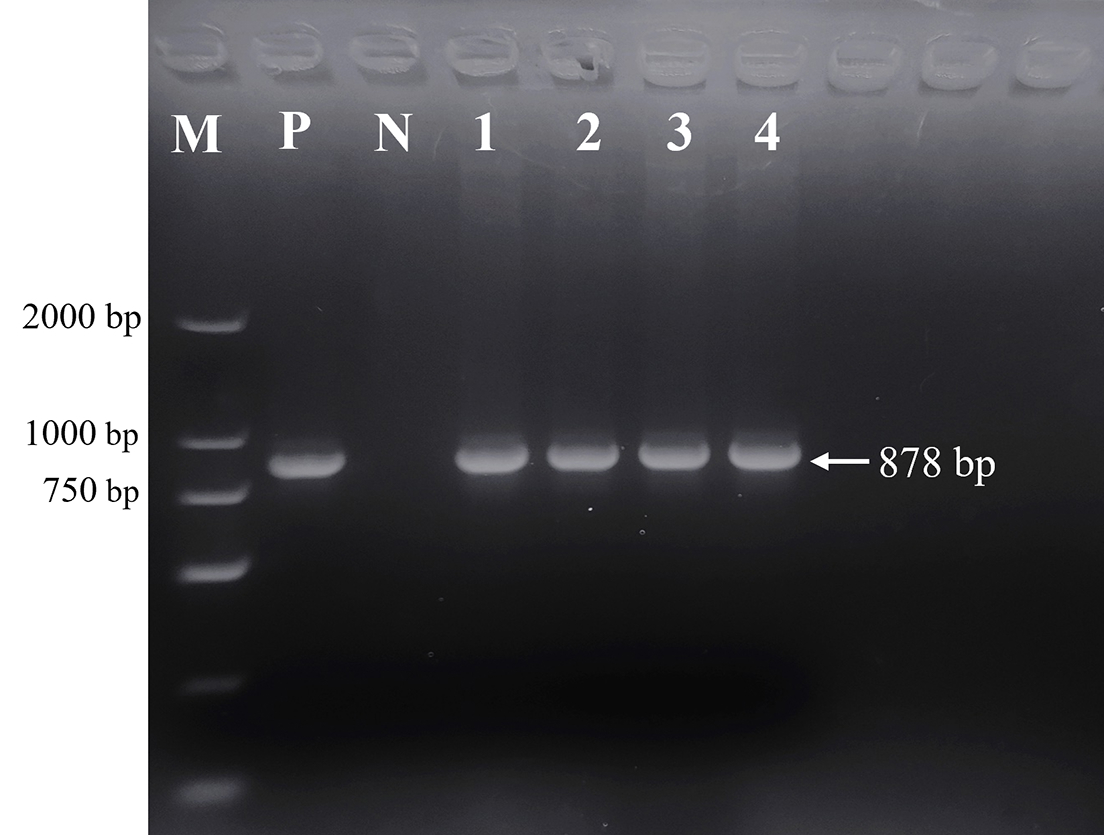


**Supplementary Figure 1.** *tem* gene detected using simplex PCR(M: Marker; P: positive control; N: negative control; 1-4: positive isolates)


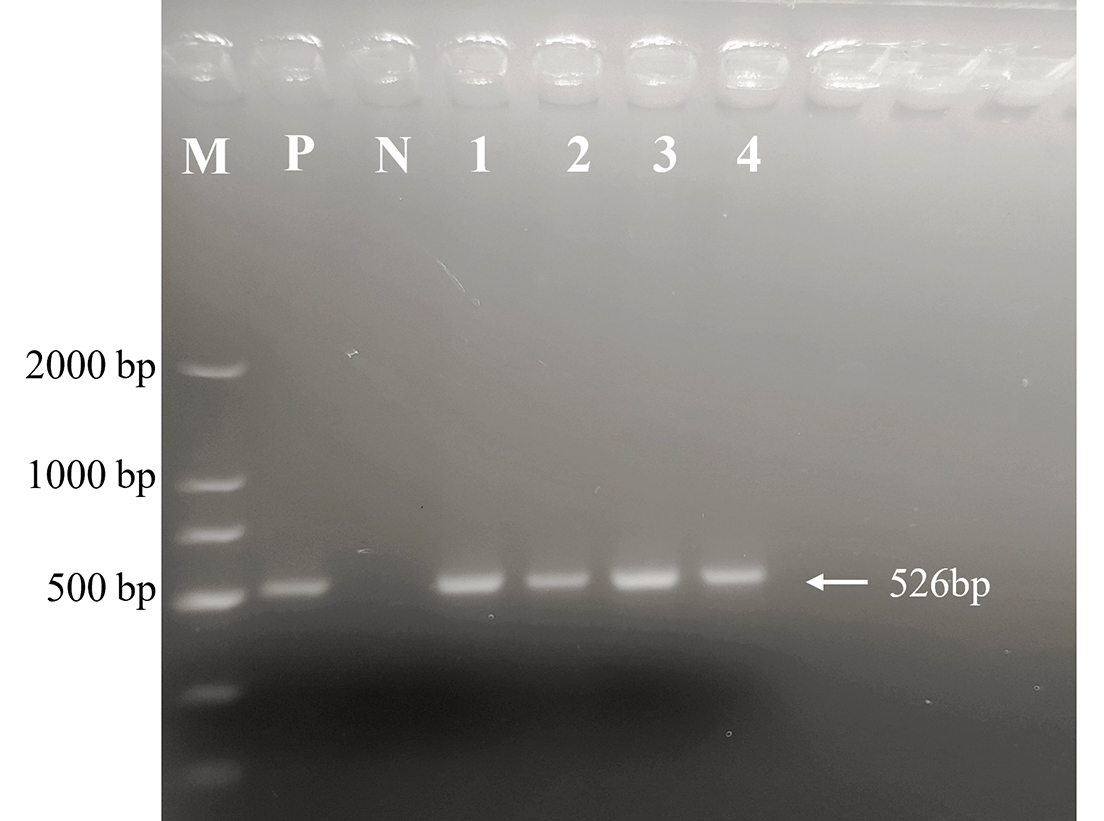


**Supplementary Figure 2.** *tem-1* gene detected using simplex PCR(M: Marker; P: positive control; N: negative control; 1-4: positive isolates)


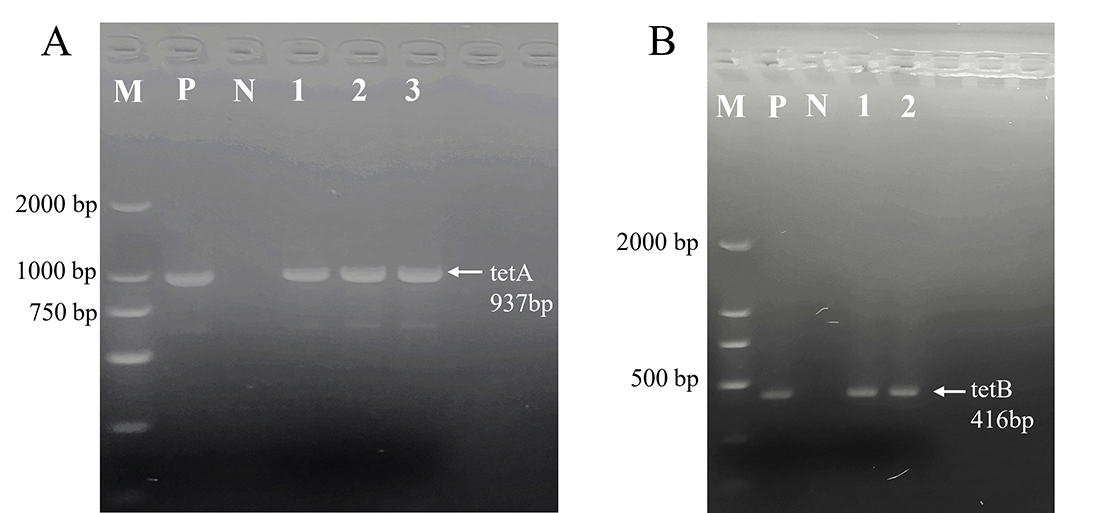


**Supplementary Figure 3.** Determination of tetracycline resistance genes (**A**) *tetA* (937 bp) and (**B**) *tetB* (416 bp)) by PCR (M: Marker; P: positive control; N: negative control; 1-4: positive isolates)


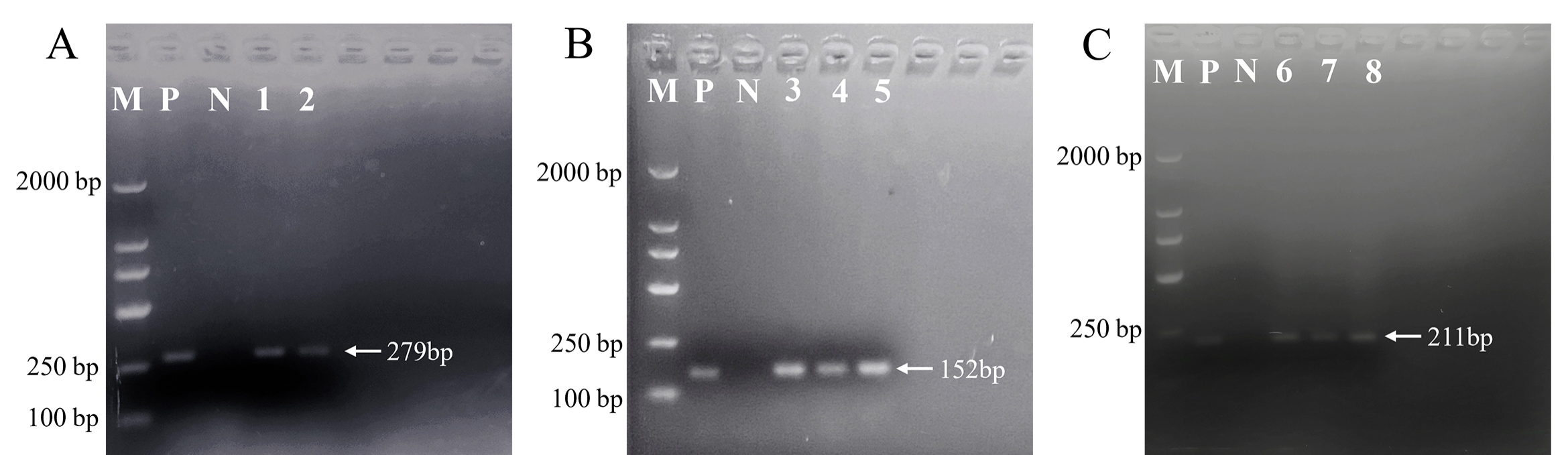


**Supplementary Figure 4.** Determination of Phylogenetic clustering genes (**A**) *chuA* (279 bp), (**B**) *TspE4.C2* (152 bp) and (**C**) *yiaA* (211 bp)) by PCR (M: Marker; P: positive control; N: negative control; 1-8: positive isolates)

**Supplementary Table 1.** Resistance gene primers and annealing temperature

| Antibiotic group | Primer | Nucleotide sequence(5′–3′) | Amplicon size (bp) | Tm (ºC) |
| --- | --- | --- | --- | --- |
| Beta-lactam | bla-TEM-1-F | TGGGTGCACGAGTGGGTTAC | 526 | 61 |
|  | bla-TEM-1-R | TTATCCGCCTCCATCCAGTC |  |  |
|  | bla-TEM-F | ATGAGTATTCAACATTTCCGTG | 861 | 56 |
|  | bla-TEM-R | TTACCAATGCTTAATCAGTGAG |  |  |
|  | bla_CTX-M-F | TTTGCGATGTGCAGTACCAGTAA | 522 | 51 |
|  | bla_CTX-M-R | CGATATCGTTGGTGGTGCCATA |  |  |
|  | bla_CTX-M9-F | GCTGGAGAAAAGCAGCGGAG | 454 | 61 |
|  | bla_CTX-M9-R | GTAAGCTGACGCAACGTCTG |  |  |
|  | cmy-2-F | AAATCGTTATGCTGCGCTCTGCTG | 1250 | 57 |
|  | cmy-2-R | TATCGGCTTTACCCCAGGTGAAA |  |  |
|  | bla-ROB-F | TCTCCCTGTTAGCCACCCTG | 574 | 61 |
|  | bla-ROB -R | CCACTGCAGCAGCTGCCGTT |  |  |
|  | oxa-23-F | GATCGGATTGGAGAACCAGA | 501 | 52 |
|  | oxa-23-R | ATTTCTGACCGCATTTCCAT |  |  |
|  | oxa-58-F | AAGTATTGGGGCTTGTGCTG | 353 | 52 |
|  | oxa-58-R | CCCCTCTGCGCTCTACATAC |  |  |
|  | pse-F | GAAAATGGGGGAGGACCGTA | 250 | 55 |
|  | pse-R | CTTAGATAATGCCGCCGAAT |  |  |
| Aminoglycoside | aph(3')-Ⅰ-F | ATGGGCTCGCGATAATGTC | 600 | 58 |
|  | aph(3')-Ⅰ-R | CTCACCGAGGCAGTTCCAT |  |  |
|  | aph(3')-ⅠA -F | TCTGAAACATGGCAAAGGTAG | 582 | 54 |
|  | aph(3')-ⅠA -R | AGCCGTTTCTGTAATGAAGGA |  |  |
|  | aac(3)-ⅡD-F | ACTGTGATGGGATACGCGTC | 237 | 60 |
|  | aac(3)-ⅡD-R | CTCCGTCAGCGTTTCAGCTA |  |  |
|  | aacA3-F | TTCATGTCCGCGAGCACCCC | 178 | 55 |
|  | aacA3-R | GACTCTTCCGCCATCGCTCT |  |  |
|  | aac(6')-ⅠB-F | ATGACTGAGCATGACCTTGC | 519 | 54 |
|  | aac(6')-ⅠB-R | TTAGGCATCACTGCGTGTTC |  |  |
|  | aadA1-F | TTTGCTGGTTACGGTGAC | 499 | 56 |
|  | aadA1-R | GCTCCATTGCCCAGTCG |  |  |
|  | aadB-F | ATGGACACAACGCAGGTCAC | 534 | 59 |
|  | aadB-R | TTAGGCCGCATATCGCGACC |  |  |
|  | strA-F | TAAAGTATGCCGTGTTCGTCT | 208 | 57.5 |
|  | strA-R | CACGCCATACTTGGAACGAG |  |  |
|  | strB-F | CTGTTCTCATTGCGGACACCT | 552 | 59.3 |
|  | strB-R | CAAAGCCCACTTCACCGACCA |  |  |
|  | aph(2'')-IF-F | TGATATATCTATATTGGGGTA | 300 | 46 |
|  | aph(2'')-IF-R | CTGCCATCAAATATAGTCG |  |  |
|  | RmtD-F | CGATTCCACAGGCAGCCGTTC | 226 | 61.5 |
|  | RmtD-R | CGACGTTTGCCCGGACACGAT |  |  |
| Tetracycline | tetA-F | GTAATTCTGAGCACTGTCGC | 937 | 62 |
|  | tetA-R | CTGCCTGGACAACATTGCTT |  |  |
|  | tetB-F | CTCAGTATTCCAAGCCTTTG | 416 | 57 |
|  | tetB-R | CTAAGCACTTGTCTCCTGTT |  |  |
|  | tetD-F | ATTACACTGCTGGACGCGAT | 1104 | 57 |
|  | tetD-R | CTGATCAGCAGACAGATTGC |  |  |
|  | tetW-F | GAGAGCCTGCTATATGCCAGC | 168 | 64 |
|  | tetW-R | GGGCGTATCCACAATGTTAAC |  |  |
|  | tetM-F | CCAAAGATAACCATGACCGAT | 853 | 53.5 |
|  | tetM-R | CAATAGATGCCCAAAACGGAT |  |  |
| Glycopeptide | vanC2-F | CGGGGAAGATGGCAGTAT | 484 | 54 |
|  | vanC2-R | CGCAGGGACGGTGATTTT |  |  |
|  | vanD-F | TGAATTGTCAGGCATTCCGTA | 509 | 56.3 |
|  | vanD-R | TCCGGTAAATCTTCATTGCT |  |  |
| Fluoroquinolone | oqxA-F | TTCTCCCCCGGCGGGAAGTAC | 162 | 57 |
|  | oqxA-R | GGCAGCGACCTTATTGGGAT |  |  |
|  | qnrS1-F | ACGACATTCGTCAACTGCAA | 212 | 57 |
|  | qnrS1-R | CGATTACTCACTTGATGGGC |  |  |
|  | gyrA-F | CGCGTACTATACGCCATGAACG | 441 | 58 |
|  | gyrA-R | ACCGTTGATCACTTCGGTCAGG |  |  |
|  | gyrB-F | GTCGTCGGCAAAATTATCGAC | 498 | 55 |
|  | gyrB-R | TTCCTGCTTACCTTTCTTCAC |  |  |
|  | aac(6')-IB-CR-F | TTGCGATGCTCTATGAGTGGCTA | 482 | 55 |
|  | aac(6')-IB-CR-R | CTCGAATGCCTGGCGTGTTT |  |  |
| Amide alcohols/ phenicols | cmlA1-F | TGTCATTTACGGCATACTCG | 455 | 55 |
|  | cmlA1-R | ATCAGGCATCCCATTCCCAT |  |  |
|  | FloR-F | AATTCCCTTTTATGGACGTT | 376 | 50.9 |
|  | FloR-R | AGGCATTGATATTTGAACACC |  |  |
|  | cat-F | CTTGTCGCCTTGCGTATAAT | 508 | 53 |
|  | cat-R | ATCCCAATGGCATCGTAAAG |  |  |
| macrolide | ereB-F | AGAAATGGAGGTTCATACTTACCA | 738 | 50 |
|  | ereB-R | CATATAATCATCACCAATGGCA |  |  |
| sulfonamide | sul1-F | GGCTGGTGGTTATGCACTCA | 263 | 61 |
|  | sul1-R | GCAGACCAATAGCGGAAGC |  |  |
|  | sul2-F | TTCGGCATCGTCAACATAACCT | 727 | 56-59 |
|  | sul2-R | CGTGTGTGCGGATGAAGTCAG |  |  |
|  | sul3-F | AGATGTGATTGATTTGGGAGC | 443 | 56 |
|  | sul3-R | TAGTTGTTTCTGGATTAGAGCCT |  |  |
| polypeptide | mcr-1-F | CGGTCAGTCCGTTTGTTC R: | 300 | 55 |
|  | mcr-1-R | CTTGGTCGGTCTGTAGGG |  |  |
